# TAT-Gap19 and Carbenoxolone Alleviate Liver Fibrosis in Mice

**DOI:** 10.3390/ijms19030817

**Published:** 2018-03-12

**Authors:** Sara Crespo Yanguas, Tereza C. da Silva, Isabel V. A. Pereira, Joost Willebrords, Michaël Maes, Marina Sayuri Nogueira, Inar Alves de Castro, Isabelle Leclercq, Guilherme R. Romualdo, Luís F. Barbisan, Luc Leybaert, Bruno Cogliati, Mathieu Vinken

**Affiliations:** 1 Department of In Vitro Toxicology and Dermato-Cosmetology, Vrije Universiteit Brussel, 1090 Brussels, Belgium; Sara.Crespo.Yanguas@vub.be (S.C.Y.); Joost.Willebrords@vub.be (J.W.); Michael.MC.Maes@vub.be (M.M.); 2 Department of Pathology, School of Veterinary Medicine and Animal Science, University of São Paulo, São Paulo 05508-270, Brazil; terezacs@usp.br (T.C.d.S.); isabelveloso@gmail.com (I.V.A.P.); bcogliati@usp.br (B.C.); 3 Department of Food and Experimental Nutrition, Faculty of Pharmaceutical Sciences, University of São Paulo, São Paulo 05508-000, Brazil; masayuri.nogueira@gmail.com (M.S.N.); inar@usp.br (I.A.d.C.); 4 Laboratory of Hepatogastroenterology, Institut de Recherche Expérimentale et clinique, Université Catholique de Louvain, 1200 Brussels, Belgium; isabelle.leclercq@uclouvain.be; 5 Department of Pathology, Botucatu Medical School, UNESP-São Paulo State University, Botucatu 18600-000, Brazil; romualdo.gr15@gmail.com (G.R.R.); barbisan@ibb.unesp.br (L.F.B.); 6 Department of Basic Medical Sciences, Physiology Group, Ghent University, 9000 Ghent, Belgium; Luc.Leybaert@UGent.be

**Keywords:** connexin43, hemichannel, gap junction, hepatic stellate cells, inflammation, liver fibrosis

## Abstract

Although a plethora of signaling pathways are known to drive the activation of hepatic stellate cells in liver fibrosis, the involvement of connexin-based communication in this process remains elusive. Connexin43 expression is enhanced in activated hepatic stellate cells and constitutes the molecular building stone of hemichannels and gap junctions. While gap junctions support intercellular communication, and hence the maintenance of liver homeostasis, hemichannels provide a circuit for extracellular communication and are typically opened by pathological stimuli, such as oxidative stress and inflammation. The present study was set up to investigate the effects of inhibition of connexin43-based hemichannels and gap junctions on liver fibrosis in mice. Liver fibrosis was induced by administration of thioacetamide to Balb/c mice for eight weeks. Thereafter, mice were treated for two weeks with TAT-Gap19, a specific connexin43 hemichannel inhibitor, or carbenoxolone, a general hemichannel and gap junction inhibitor. Subsequently, histopathological analysis was performed and markers of hepatic damage and functionality, oxidative stress, hepatic stellate cell activation and inflammation were evaluated. Connexin43 hemichannel specificity of TAT-Gap19 was confirmed in vitro by fluorescence recovery after photobleaching analysis and the measurement of extracellular release of adenosine-5′-triphosphate. Upon administration to animals, both TAT-Gap19 and carbenoxolone lowered the degree of liver fibrosis accompanied by superoxide dismutase overactivation and reduced production of inflammatory proteins, respectively. These results support a role of connexin-based signaling in the resolution of liver fibrosis, and simultaneously demonstrate the therapeutic potential of TAT-Gap19 and carbenoxolone in the treatment of this type of chronic liver disease.

## 1. Introduction

Liver fibrosis is the result of a coordinated and conserved interplay between resident hepatic cells, infiltrating inflammatory cells, and a number of paracrine and autocrine signals that lead to the transdifferentiation of quiescent hepatic stellate cells (HSCs) into activated myofibroblast-like counterparts [1,2]. Among these signaling cascades, inflammatory responses have gained increased attention in the last few years. Hepatic macrophages, monocytes, natural killer T cells, and B cells release a number of factors, such as reactive oxygen species, tumor necrosis factor α, platelet-derived growth factor, transforming growth factor β, and other cytokines, which promote HSC activation [3,4]. Communication mechanisms that are participating in inflammation-promoting hepatic fibrosis have been much less documented thus far. Direct intercellular communication is predominantly mediated by gap junctions (GJs), which allow the flux of hydrophilic molecules up to ~1.5 kDa, such as adenosine-5′-triphosphate (ATP) and ions between adjacent cells [5,6,7]. GJs arise from the docking of two hemichannels from neighboring cells, each consisting of six connexin (Cx) proteins. While GJs are involved in both physiological and pathological processes, hemichannels seem to be consistently associated with stress conditions [8,9,10]. At present, more than 20 Cx species have been identified in mammals and are named after their predicted molecular weight expressed in kDa. In rodent and human liver, Cx43 is mainly harbored by nonparenchymal cells, including HSCs, Kupffer cells, endothelial cells, and cholangiocytes, while Cx32, and to a lesser extent, Cx26, are abundantly expressed by hepatocytes [9]. Hepatic Cx expression patterns undergo drastic changes in a broad range of liver diseases, such as acute liver injury [11], hepatitis [12], cholestasis [13], cirrhosis [14], and hepatocellular carcinoma [15]. Typically, an upregulation of Cx43 production is observed, while Cx32 expression is negatively affected in liver disease [16]. In liver fibrosis, increased amounts of Cx43 have been detected in activated HSCs [17] and activated Kupffer cells [18]. In addition, infiltrating immune cells also contribute to the overall hepatic increase in Cx43 expression [18]. The role of enhanced Cx43 production, in particular in activated HSCs, is not clear. In cultures of activated HSCs, the chemical inhibition of Cx43-based GJs triggered a reduction in cellular contractibility [17]. Furthermore, Cx43 deficient-mice developed excessive scarring with reduced inflammatory cell infiltration and hepatocellular damage in liver fibrosis [19], suggesting a protective effect of Cx43-based signaling against hepatic scar formation, as well as a role in the control of cell death and inflammation. The present study was set up to further investigate the involvement of connexin43 signaling, through both hemichannels and GJs, in liver fibrosis. In order to do so, a number of channel inhibitors were used, namely carbenoxolone (CBX) and Gap19. While CBX is a general blocker of hemichannel and GJ signaling [20,21], Gap19 specifically inhibits Cx43 hemichannels, thereby leaving GJs unaffected [22]. Since the target of Gap19 is located intracellularly, it was linked to a HIV transactivator of transcription (TAT) sequence in order to facilitate the passage through the plasma membrane [22,23].

## 2. Results

### 2.1. Effects of CBX and TAT-Gap19 on Gap Junctions and Hemichannels in Cultures of Primary Rat Hepatocytes

**Figure 1:**
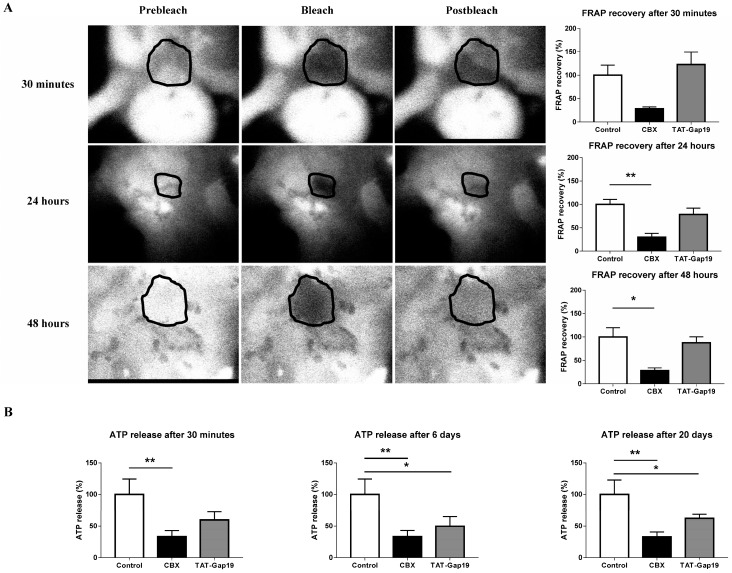
Effects of carbenoxolone (CBX) and transactivator of transcription (TAT)-Gap19 on gap junctions (GJs), and hemichannels in cultures of primary rat hepatocytes. Primary rat hepatocytes were exposed to 50 μM CBX, 20 μM TAT-Gap19, or vehicle control. (**A**) GJ activity was measured through FRAP analysis after 30 min, 24 h and 48 h (*n* = 4, *N* = 4). (**B**) Hemichannel activity was determined by measurement of extracellular ATP release analysis after 30 min (*n* =3, *N* =6). TAT-Gap19 was incubated at 37 °C for 0 min, 6 days, and 20 days in an incubator prior to functionality assessment. Results were analyzed by 1-way ANOVA followed by post hoc Bonferroni correction. Data were expressed as means ± SEM (* *p* ≤ 0.05; ** *p* ≤ 0.01).

To confirm the channel specificity of TAT-Gap19 in a hepatic system, fluorescence recovery after photobleaching (FRAP) analysis and measurement of extracellular release of ATP were performed in cultures of primary rat hepatocytes (Figure 1). In contrast to the in vivo situation, cultured primary rat hepatocytes express Cx43-based hemichannels and gap junctions [24], which makes them a suitable model to investigate TAT-Gap19 target specificity. Specifically, 24 h after cell plating, primary rat hepatocytes were exposed to 50 μM CBX, 20 μM TAT-Gap19, or vehicle control for 30 min, 24 h and 48 h. FRAP analysis revealed that GJ inhibition occurred when hepatocytes were exposed to CBX for 24 h (*p* ≤ 0.01) and 48 h (*p* ≤ 0.05). In contrast, no modification in fluorescence recovery was noticed after exposure to TAT-Gap19 measured on these time points (Figure 1A). To investigate the effects of both CBX and TAT-Gap19 on hemichannel communication, a divalent-free (DF) buffer was used as a trigger of hemichannel opening followed by measurement of extracellular ATP amounts. To indirectly test the stability, TAT-Gap19 was incubated at 37 °C for 0 min, 6 days, and 20 days in a classic incubator prior to the cell culture testing. Primary rat hepatocytes were exposed to 50 μM CBX, 20 μM TAT-Gap19, or vehicle control for 30 min. TAT-Gap19 was found to significantly inhibit extracellular ATP release incubation at 37 °C for 6 days (*p* ≤ 0.05) and 20 days (*p* ≤ 0.05). CBX suppressed extracellular liberation of ATP at all measured time points (*p* ≤ 0.01) (Figure 1B). These results confirm the selectivity of TAT-Gap19 to block hemichannels and not GJs.

### 2.2. Effects of CBX and TAT-Gap19 on the Fibrotic Response after TAA-Induced Chronic Hepatic Injury in Mice

**Figure 2:**
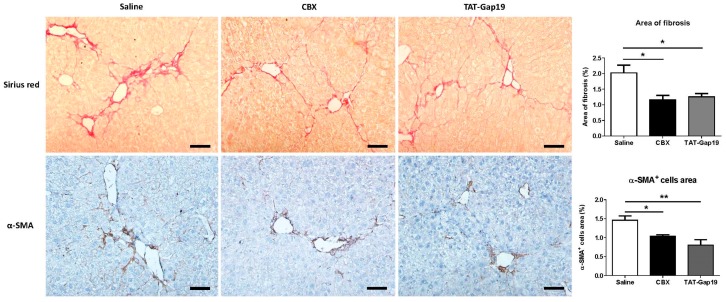
Effects of CBX and TAT-Gap19 on the fibrotic response after thioacetamide (TAA)-induced chronic hepatic injury in mice. Mice were administered TAA intraperitoneally for eight weeks. Initially, mice (*n* = 5/group) were administered 100 mg TAA/kg body weight, followed by 10% weekly increments to the utmost dose of approximately 200 mg TAA/kg body weight. Thereafter, an osmotic pump was implanted in the peritoneal cavity, which ensured the release of 1 mg TAT-Gap19 or CBX/kg body weight/day or saline for two weeks. Collagen morphometric analysis was performed by the quantification of the area of collagen fibers stained by Sirius red. Hepatic stellate cells (HSC) activation was assessed through immunohistochemistry analysis of α-SMA-positive cells area. At least 10 randomly selected fields were quantified from the left lobe of each animal. Results were analyzed by 1-way ANOVA followed by *post hoc* Bonferroni correction. Data were expressed as means ± SEM (* *p* ≤ 0.05; ** *p* ≤ 0.01). Scale bar represents 50 μm.

**Figure A1:**
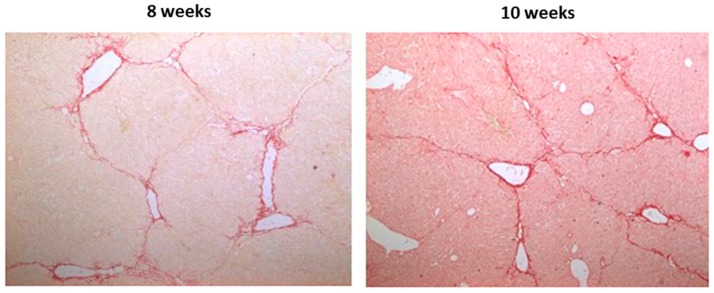
TAA-induced liver fibrosis in mice before and after withdrawal of the insult. Mice were administered TAA intraperitoneally for 8 weeks. Initially, mice were administered with 100 mg TAA/kg body weight, followed by 10% weekly increments to the utmost dose of approximately 200 mg TAA/kg body weight. Thereafter, mice were not treated with TAA for 2 weeks (10 weeks in total). Collagen deposition was visualized by collagen fibers stained with Sirius red.

To ensure a constant delivery of 1 mg/kg body weight/day of the channel inhibitors, TAT-Gap19, and CBX were administered through an osmotic pump implanted in the peritoneal cavity of mice subjected to treatment with 100–200 mg thioacetamide (TAA)/kg body weight for eight weeks. Two weeks after osmotic pump implantation, the outcome on liver fibrosis was evaluated. In particular, hepatic collagen content, and thus the degree of fibrosis, was assessed through the measurement of the area of collagen staining of liver sections with Sirius red and concomitant determination of the percentage of collagen per section, while the activated HSC quantities were evaluated through immunohistochemistry analysis of the area of alpha smooth muscle actin (α-SMA)-positive cells and subsequent quantification of the percentage of the area of α-SMA-positive cells per section. Initially, and as previously confirmed by others [25], no changes in the collagen deposition were observed as a consequence of the withdrawal of the insult (Figure A1). TAT-Gap19-treated mice showed significantly decreased collagen deposition (*p* ≤ 0.05), as well as lowed amounts of α-SMA-positive cells area (*p* ≤ 0.01). A reduced collagen content (*p* ≤ 0.05) and lower quantities of α-SMA-positive cells area (*p* ≤ 0.05) were equally found in CBX-treated mice (Figure 2), with no differences between the treated groups. These data suggest that communication mediated by Cx43-based hemichannels and GJs play a crucial role in the maintenance of the fibrotic response.

### 2.3. Effects of CBX and TAT-Gap19 on Biochemical Parameters after TAA-Induced Chronic Hepatic Injury in Mice

**Figure 3:**
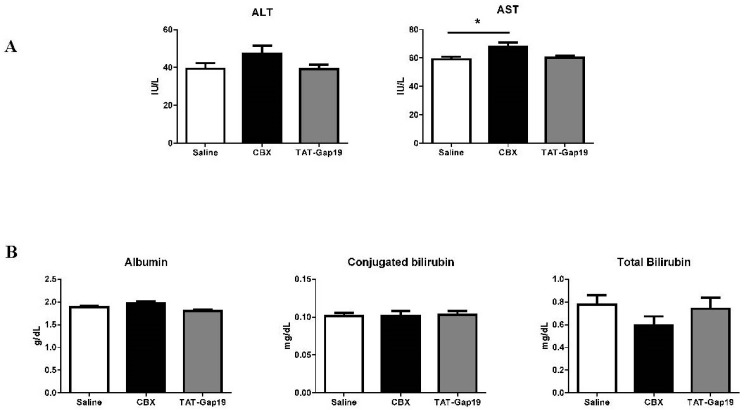
Effects of CBX and TAT-Gap19 on biochemical parameters after TAA-induced chronic hepatic injury in mice. Mice were administered TAA intraperitoneally for 8 weeks. Initially, mice (*n* = 15/group) were administered with 100 mg TAA/kg body weight, followed by 10% weekly increments to the utmost dose of approximately 200 mg TAA/kg body weight. Thereafter, an osmotic pump was implanted in the peritoneal cavity, which ensured the release of 1 mg TAT-Gap19 or CBX/kg body weight/ or saline for two weeks. (**A**) Serum levels of the necrotic cell death markers namely ALT and AST, (**B**) Serum levels of the hepatic functionality markers albumin, conjugated and total bilirubin. Results were analyzed by 1-way ANOVA followed by post hoc tests Bonferroni correction. Data were expressed as means ± SEM (* *p* ≤ 0.05).

TAT-Gap19 did not affect serum levels of the necrotic cell death markers alanine aminotransferase (ALT) and aspartate aminotransferase (AST). Surprisingly, CBX increased AST quantities (*p* ≤ 0.05). Neither TAT-Gap19 nor CBX influenced serum levels of albumin and conjugated and total bilirubin, which is indicative of hepatocyte functionality (Figure 3).

### 2.4. Effects of CBX and TAT-Gap19 on Anti-Oxidative Enzyme Activity after TAA-Induced Chronic Hepatic Injury in Mice

**Figure 4:**
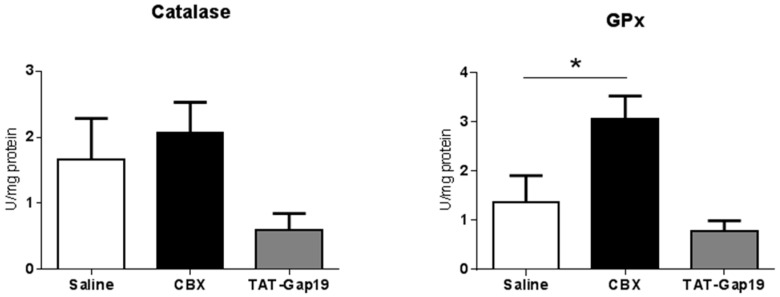

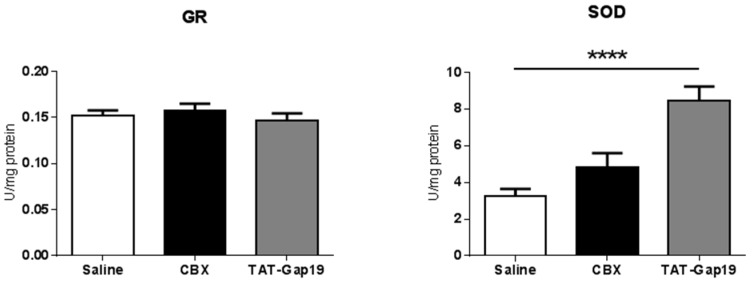
Effects of CBX and TAT-Gap19 on anti-oxidative enzyme activity after TAA-induced chronic hepatic injury in mice. Mice were administered TAA intraperitoneally for 8 weeks. Initially, mice (*n* = 8/group) were administered with 100 mg TAA/kg body weight, followed by 10% weekly increments to the utmost dose of approximately 200 mg TAA/kg body weight. Thereafter, an osmotic pump was implanted in the peritoneal cavity, which ensured the release of 1 mg TAT-Gap19 or CBX/kg body weight/day or saline for two weeks. The activity of the anti-oxidant enzymes catalase, glutathione peroxidase (GPx), glutathione reductase (GR), and superoxide dismutase (SOD) was evaluated in liver tissue. Results were analyzed by 1-way ANOVA followed by post hoc Bonferroni correction. Data were expressed as means ± SEM (* *p* ≤ 0.05; **** *p* ≤ 0.0001).

Oxidative stress with generation of free radicals and lipid peroxidation constitutes a major contributor to the progression of liver fibrosis [26]. For this reason, the activity of a number of oxidative stress scavengers, including catalase, glutathione peroxidase (GPx), glutathione reductase (GR), and superoxide dismutase (SOD), was assessed on liver tissue of fibrotic mice. TAT-Gap19-treated mice showed increased SOD activity (*p* ≤ 0.0001) and no changes in catalase, GPx and GR activities. Upon CBX treatment, the activity of GPx was significantly upregulated (*p* ≤ 0.05), with catalase, GR, and SOD being unmodified (Figure 4). These results indicate enhanced anti-oxidative stress defence as a result of TAT-Gap19 and CBX treatments.

### 2.5. Effects of CBX and TAT-Gap19 on the Inflammatory Response after TAA-Induced Chronic Hepatic Injury in Mice

**Figure 5:**
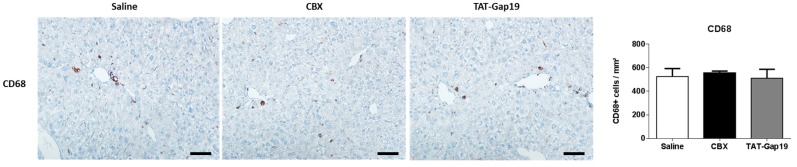
Effects of CBX and TAT-Gap19 on the inflammatory response after TAA-induced chronic hepatic injury in mice. Mice were administered TAA intraperitoneally for 8 weeks. Initially, mice (*n* = 5/group) were administered with 100 mg TAA/kg body weight, followed by 10% weekly increments to the utmost dose of approximately 200 mg TAA/kg body weight. Thereafter, an osmotic pump was implanted in the peritoneal cavity, which ensured the release of 1 mg TAT-Gap19 or CBX/kg body weight/day or saline for two weeks. Macrophage abundance was determined by quantification of CD68 immunostaining in liver tissue. At least 10 randomly selected fields were quantified from the left lobe of each animal. Results were analyzed by 1-way ANOVA followed by post hoc Bonferroni correction. Data were expressed as means ± SEM. Scale bar represents 50 μm.

Immune cells contribute to the liver fibrotic response by promoting hepatocellular damage and HSC activation and also participate in fibrosis regression by extracellular matrix degradation [27]. Macrophages are believed to be the main mediators in both processes [28]. Accordingly, the number of macrophages was evaluated through immunostaining of CD68 in liver tissue. CD68-positive cells were mainly located in the sinusoids and in the surrounding area of the scar tissue. Importantly, TAT-Gap19 and CBX did not affect CD68-positive cell density or location (Figure 5).

**Figure 6:**
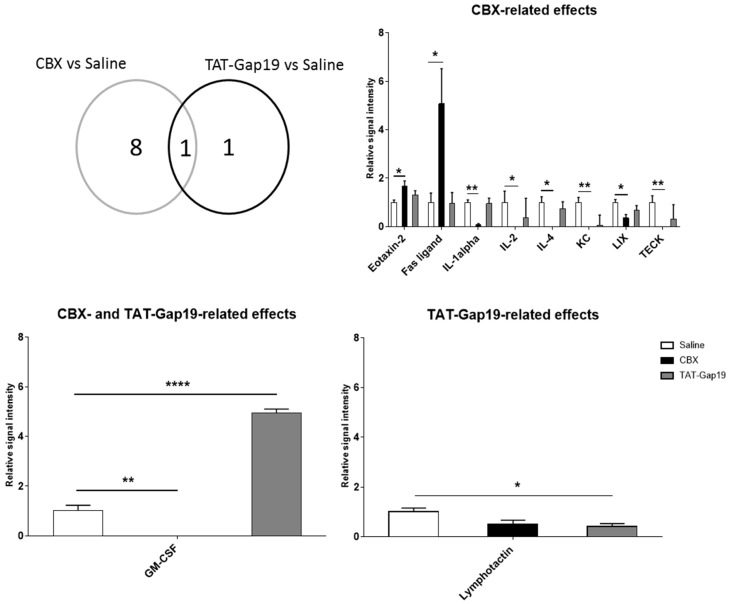
Effects of CBX and TAT-Gap19 on the inflammatory response after TAA-induced chronic hepatic injury in mice. Mice were administered TAA intraperitoneally for eight weeks. Initially, mice (*n* = 4/group) were administered with 100 mg TAA/kg body weight, followed by 10% weekly increments to the utmost dose of approximately 200 mg TAA/kg body weight. Thereafter, an osmotic pump was implanted in the peritoneal cavity, which ensured the release of 1 mg TAT-Gap19 or CBX/kg body weight/day or saline for two weeks. After comparison between CBX versus saline with TAT-Gap19 versus saline groups, three clusters of inflammatory proteins were defined, namely (i) CBX-related effects; (ii) CBX- and TAT-Gap19-related effects; and (iii) TAT-Gap19-related effects. Data were analyzed by densitometric analysis. Then, background was subtracted, and results were normalized against the average of positive controls. Results were analyzed by 1-way ANOVA followed by post hoc Bonferroni correction. Data were expressed as means ± SEM (* *p* ≤ 0.05; ** *p* ≤ 0.01; **** *p* ≤ 0.0001). (GM-CSF, granulocyte-macrophage colony-stimulating factor; IL1α/2/4, interleukin 1α/2/4; KC, growth-regulated alpha protein; LIX, lipopolysaccharide-induced CXC chemokine).

**Figure A2:**
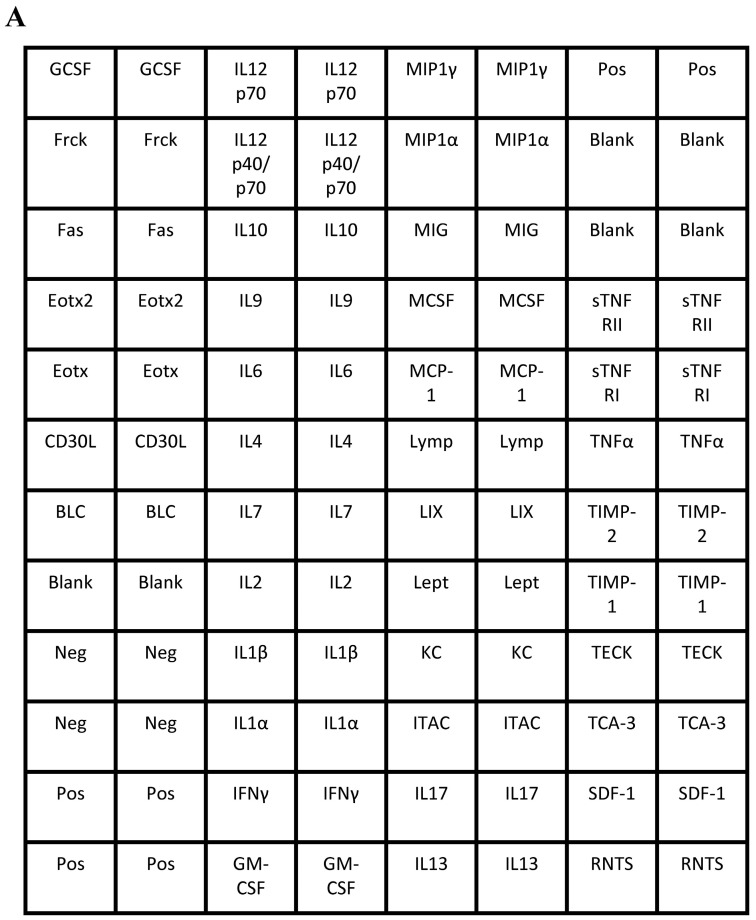

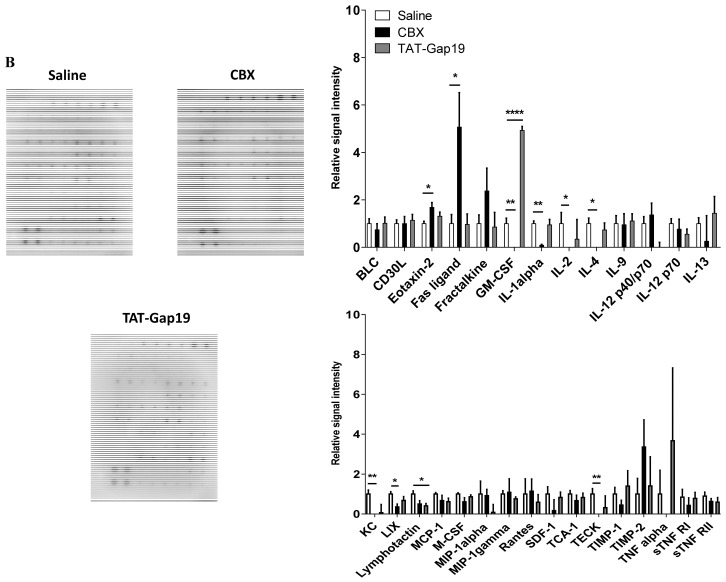
Effects of CBX and TAT-Gap19 on the inflammatory response after TAA-induced chronic hepatic injury in mice. Mice were administered TAA intraperitoneally for 8 weeks. Initially, mice (*n* = 4/group) were administered with 100 mg TAA/kg body weight, followed by 10% weekly increments to the utmost dose of approximately 200 mg TAA/kg body weight. Thereafter, an osmotic pump was implanted in the peritoneal cavity, which ensured the release of 1 mg TAT-Gap19 or CBX/kg body weight/day or saline for 2 weeks. (**A**) Antibody array map with the inflammatory proteins. (**B**) Representative antibody array membranes from mouse liver tissue extract corresponded to animals treated with saline, CBX or TAT-Gap19. Data were analyzed by densitometric analysis. Then, background was subtracted and results were normalized against the average of positive controls. Results were analyzed by 1-way ANOVA followed by post hoc Bonferroni correction. Data were expressed as means ± SEM (* *p* ≤ 0.05; ** *p* ≤ 0.01; **** *p* ≤ 0.0001). (BLC, B lymphocyte chemoattractant; CD30L, tumor necrosis factor ligand superfamily member 8; Eotx, eotaxin; Frck, fractalkine, GCSF, granulocyte colony-stimulating factor; GM-CSF, granulocyte-macrophage colony-stimulating factor; IFNγ, interferon γ; IL1α/2/7/4/6/9/10/12/13/17, interleukin 1 alpha/2/7/4/6/9/10/12/13/17; ITAC, interferon-inducible T-cell α chemoattractant; KC, growth-regulated α protein; LIX, lipopolysaccharide-induced CXC chemokine; Lymp, lymphotactin; MCP-1, monocyte chemoattractant protein 1; MCSF, macrophage colony-stimulating factor; MIG, monokine induced by gamma interferon; MIP1α/γ, macrophage inflammatory protein 1 alpha/gamma; Neg, negative; Pos, positive; RNTS, rantes; SDF-1, stromal cell derived factor 1; sTNF RI/II, soluble TNF receptor 1/2; TCA-3, T-cell activation protein 3; TECK, thymus-expressed chemokine; TIMP-1/-2, tissue inhibitor of metalloproteinase-1/-2; TNFα, tumor necrosis factor α).

Further evaluation of inflammatory protein levels by semi-quantitative antibody array analysis of liver tissue showed differential effects of TAT-Gap19 and CBX treatments (Figure A2). TAT-Gap19-treated mice displayed a downregulation of lymphotactin (*p* ≤ 0.05) (Figure 6). In addition, CBX-treated mice presented increased protein levels of eotaxin-2 (*p* ≤ 0.05) and Fas ligand (*p* ≤ 0.05), whereas a reduction in interleukin 1α (*p* ≤ 0.01), 2 (*p* ≤ 0.05), and 4 (*p* ≤ 0.05) (IL1α/2/4), growth-regulated alpha protein (KC) (*p* ≤ 0.01), lipopolysaccharide-induced CXC chemokine (LIX) (*p* ≤ 0.05), and thymus-expressed chemokine (TECK) (*p* ≤ 0.01) was observed (Figure 6). Interestingly, upon comparison between CBX- and TAT-Gap19-related effects, granulocyte-macrophage colony-stimulating factor (GM-CSF) was commonly modulated. In this context, TAT-Gap19-treated mice showed an upregulation of GM-CSF (*p* ≤ 0.0001), whereas GM-CSF levels were downregulated (*p* ≤ 0.01) in CBX-treated animals. These results point to the modulation of the inflammatory response as a consequence of CBX treatment.

## 3. Discussion

In-depth knowledge of the molecular mechanisms that regulate liver fibrosis is of clinical importance for the development of new therapies. In the last decade, it has become clear that Cx hemichannels can act as pathological pores. Indeed, their inhibition counteracts the manifestation of liver disease, including acute liver failure [29] and non-alcoholic steatohepatitis [30]. In the present study, it was investigated whether this also holds true for liver fibrosis by using channel inhibitors. In this regard, a number of short peptides have been introduced several years ago as selective tools to block hemichannels, thereby leaving GJ activity unaffected [20,31]. Among those is TAT-Gap19, which is designed to specifically inhibit Cx43-based hemichannels. The efficacy and specificity of TAT-Gap19 to suppress Cx43 hemichannel opening has been previously demonstrated in astrocytes [23], cardiomyocytes [22], and human gingival fibroblasts [32]. Our experiments showed that this is also the case in primary rat hepatocytes. In these experiments, TAT-Gap19 incubated for extended periods of time at 37 °C was used as a surrogate measurement of peptide stability for the in vivo study. It should be noted that the effect exerted by the treatment with TAT-Gap19 and CBX is only attributed to channel inhibition and not to altered Cx43 protein expression levels. Indeed, TAT-Gap19 was previously shown to leave Cx43 expression unaffected [30]. Similar findings were reported for CBX [24]. Administration of TAT-Gap19 as well as of the general GJ and hemichannel inhibitor CBX to liver fibrotic mice decreased fibrotic areas in liver and reduced HSC activation, as evidenced by lowered amounts of α-SMA-positive cells area. The mechanisms that are underpinning these effects are not clear. Although CBX has the ability to abrogate GJ intercellular communication in activated HSCs [17], studies based on in vitro-induced activation of primary HSCs showed that the effects exerted by CBX are independent of GJ inhibition [33]. In addition, expression profiles of collagens type I and III, as well as of α-SMA, are known to be affected by Cx43-based hemichannels in human gingival fibroblasts [32]. These data suggest that Cx43-based hemichannels rather than their full channel GJ counterparts are involved in the activation of HSCs. In addition, administration of TAT-Gap19 and CBX did not alter the promotion of tissue damage, with the exception of the increased AST levels observed upon CBX treatment. It should be stressed, however, that TAT-Gap19 and CBX released from the osmotic pump in the abdominal cavity not only act on liver cells, but also may equally target other organs and cells, especially those that are involved in inflammatory responses. Nevertheless, no modification in the number of CD68-positive macrophages was observed following treatment with TAT-Gap19 or CBX, yet production of inflammatory proteins was changed. Upon comparison, three inflammatory protein clusters could be identified as differentially modulated, namely (i) CBX-related effects; (ii) CBX- and TAT-Gap19-related effects; and (iii) TAT-Gap19-related effects. Specifically, CBX elicited an upregulation of Fas ligand, which is an indicator of myofibroblast apoptosis [34]. This could explain the decreased number of activated HSCs upon CBX treatment. This coincided with reduced amounts of IL1α, IL2, IL4, KC, LIX, and TECK. Among those, IL1α has been observed to be decreased in autophagic macrophages [35], which contributes to the suppression of the fibrotic response. During liver fibrosis, TECK is secreted by liver sinusoidal endothelial cells and favors HSC migration as well as activation by macrophages [36], which further supports disease regression. Hence, CBX-related signaling pathways might be involved in the modulation of immune responses-mediated hepatic fibrosis. These results should; however, be considered with caution, since CBX also affects the opening of many other channels, including pannexin1 channels [37], in addition to inhibiting Cx-based hemichannels and GJs. In both the TAT-Gap19- and CBX-treated animal groups, an opposite effect was observed on GM-CSF. While increased expression of GM-CSF, a cytokine that contributes to the differentiation of monocytes into macrophages and dendritic cells [38,39], was observed upon Cx43 hemichannel inhibition, a decreased production was seen following CBX administration. In the TAT-Gap19-treated group, only lymphotactin was modified. An alternative anti-fibrotic mechanism includes excessive oxidative stress. During activation, HSCs favor the anti-oxidant activity of GPx and catalase to prevent cellular apoptosis in the presence of hydrogen peroxide [40]. Cx43 hemichannel inhibition led to the accumulation of hydrogen peroxide due to SOD overactivation and the absence of effects on GPx and catalase activities, which may partially contribute to myofibroblast apoptosis. This is in agreement with a previous report showing that Cx43 hemichannels provide a protective mechanism against oxidative stress in osteocytes [41].

In summary, this study revealed important changes in communication mechanisms that were mediated by Cx43 during liver fibrosis, whereby the effects mediated by GJs and hemichannels might be linked to modifications in inflammatory and anti-oxidant defense, respectively. Future research should determine a more specific role of Cx hemichannels and GJs in the different liver cells during the fibrotic response.

## 4. Materials and Methods

### 4.1. Animals and Treatment

**Figure 7:**
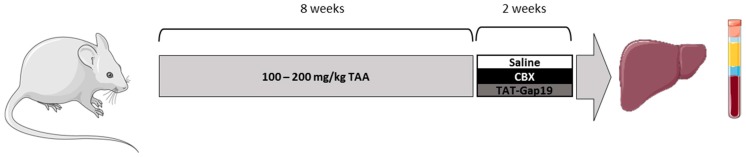
Experimental design. Mice were administered intraperitoneally with TAA for eight weeks. Initially, mice were administered with 100 mg TAA/kg body weight, followed by 10% weekly increments to the utmost dose of approximately 200 mg TAA/kg body weight. Thereafter, an osmotic pump was implanted in the peritoneal cavity, which ensured the release of 1 mg TAT-Gap19 or CBX/kg body weight/day or saline for two weeks. Mice were euthanized after completion of the treatment and blood and liver samples were collected for further analysis.

Male Balb/c mice were obtained from Jackson Laboratories (Bar Harbor, ME, USA). Animals were housed in the animal facility of the School of Veterinary Medicine and Animal Science of the University of São Paulo-Brazil. Mice were kept in a room with ventilation (i.e., 16–18 air changes/hour), relative humidity (i.e., 45–65%), controlled temperature (i.e., 20–24 °C), and light/dark cycle 12:12, and were given water and balanced diet (NUVILAB-CR1, Nuvital Nutrientes LTDA, Colombo, Brazil) ad libitum. Liver fibrosis was induced by administration of TAA (Sigma-Aldrich, St. Louis, MO, USA), which is a chemical that promotes a relatively stable fibrotic response with slow regression over time [25]. Mice were weighed (22 ± 4 g) and received three weekly doses of TAA diluted in physiological solution, administered intraperitoneally for eight weeks, as previously described, with small modifications [42]. The initial dose of TAA was 100 mg/kg body weight followed by 10% weekly increments to the utmost dose of approximately 200 mg TAA/kg body weight. Two days after final TAA dosing, mice were treated daily with 1 mg TAT-Gap19 or CBX/kg body weight. Both of the compounds were administered via an osmotic pump (Alzet, Cupertino, CA, USA) implanted in the peritoneal cavity for two weeks (Figure 7). Control mice were treated in parallel with saline. Mice were euthanized after completion of the treatment by exsanguination during sampling under isoflurane-induced anesthesia. Mouse blood, collected by cardiac puncture, was drawn into a heparinized syringe and centrifuged for 10 min at 1503× *g*, and serum was stored at −20 °C. Mouse livers were excised, and fragments were fixed in 10% phosphate-buffered formalin or snap-frozen in liquid nitrogen with storage at −80 °C. This study has been approved by the Committee on Bioethics of the School of Veterinary Medicine and Animal Science of the University of São Paulo-Brazil (protocol number 9999100314, 13 October 2015), and all of the animals received humane care according to the criteria outlined in the “Guide for the Care and Use of Laboratory Animals”.

### 4.2. Hepatocyte Rat Isolation and Cultivation

Male outbred Sprague-Dawley rats (Charles River Laboratories, Brussels, Belgium) were kept under controlled environmental conditions with free access to food and water. Hepatocyte rat isolation and cultivation procedures were performed, as previously detailed [30]. Briefly, rat hepatocytes were isolated by use of a two-step collagenase perfusion method. After purification, cell viability was assessed by trypan blue exclusion. Viable (≥85%) rat hepatocytes were plated at a density of 0.56 × 10^5^ cells per cm^2^ in William’s medium E (Invitrogen, Carlsbad, CA, USA) supplemented with 7 ng/mL glucagon, 292 mg/mL l-glutamine, antibiotics (7.33 IU of sodium benzyl penicillin, 50 μg/mL kanamycin monosulfate, 10 μg/mL sodium ampicillin, and 50 μg/mL streptomycin sulfate), and 10% fetal bovine serum. After 4, 24 and 48 h, the cell culture medium was removed and replaced by serum-free medium supplemented with 25 μg/mL hydrocortisone sodium hemisuccinate and 0.5 μg/mL insulin. This study was approved by the Ethical Committee for Animal Experiments of the Vrije Universiteit Brussel (project number 14-210-1, 3 February 2015) and all animals received humane care according to the criteria outlined in the “Guide for the Care and Use of Laboratory animals”.

### 4.3. TAT-Gap19 and CBX

TAT-Gap19 (YGRKKRRQRRR-KQIEIKKFK) was synthesized by Thermo Fischer (Schwerte, Germany) at a purity of at least 90%. For in vitro and in vivo experiments, TAT-Gap19 was dissolved in Hank’s balanced salt solution (Thermo Fisher Scientific, Waltham, MA, USA) buffered with 25 mM Hepes (HBBS-Hepes) and saline, respectively. In the same line, CBX disodium salt (Sigma-Aldrich) was dissolved in HBSS-Hepes and saline for cell culture and animal experiments, respectively.

### 4.4. Fluorescence Recovery after Photobleaching

FRAP analysis was performed 24 h after cell plating as previously described [24,30]. Cultured rat hepatocytes were exposed to 50 μM CBX, 20 μM TAT-Gap19 or vehicle control for 30 min, 24 h and 48 h. Fluorescence within a single cell was photobleached by one second spot exposure to 488 nm Argon laser light, and dye influx from neighboring cells was recorded over the next 5 min with a 40× water immersion objective (Nikon, Tokyo, Japan). Fluorescence in the bleached cell was expressed as the percentage recovery relative to the prebleach level. At least four cells per culture dish were examined.

### 4.5. Measurement of Extracellular Adenosine-5′- Triphosphate

Extracellular ATP release was measured using a commercial luciferin/luciferase kit (Sigma-Aldrich) as previously outlined [24]. TAT-Gap19 was pre-incubated at 37 °C for 0 min, 6 days, or 20 days in an incubator (Galaxy 170S, New Bruswich, Hamburg, Germany). Thereafter, primary rat hepatocyte cultures were exposed to 50 μM CBX, 20 μM TAT-Gap19 or vehicle control for 30 min. These experiments were carried out 24 h after cell plating. After exposure, cultured rat hepatocytes were washed with DF buffer (137 mM NaCl, 0.18 mM Na_2_HPO_4_·2H_2_O, 5.36 mM KCl, 0.44 mM KH_2_PO_4_, 4 mM ethylene glycol tetra-acetic acid, 5.55 mM d-glucose, and 25 mM Hepes) to trigger hemichannel opening or with HBBS-Hepes to mimic baseline conditions and incubated for 2.5 min to DF buffer or HBSS-Hepes at room temperature. Then, ATP assay mix was added, and luminescence was measured. ATP release was expressed as the percentage of ATP release triggered by the DF buffer.

### 4.6. Histopathological Liver Examination and Collagen Analysis

For microscopic evaluation, formalin-fixed liver fragments were embedded in paraffin and 5 µm sections were stained with Sirius red for blinded evaluation of the liver, as previously described [19]. Morphometric analysis of Sirius red staining was performed in 10 randomly selected fields per section from the left lobe of the mouse liver (20× objective). Semi-quantitative analysis of the fibrotic area was performed with Image-Pro Plus 4.5 software (Media Cybernetics, Silver Spring, MD, USA) and calculated by the formula [Area of fibrosis (%) = total fibrosis area/total area of the liver tissue].

### 4.7. Immunohistochemistry

Immunoreactivity for α-SMA and CD68 was performed on liver mouse sections. Briefly, 5 µm mouse liver sections were subjected to antigen retrieval at 120 °C for 5 min in a Pascal Pressure Chamber (Dako Cytomation, Glostrup, Denmark) and blocked for 15 min. Then, slides were treated with low-fat milk for 60 min and incubated in a humidified chamber overnight at 4 °C with 1/500 anti-α-SMA (Abcam, Cambridge, UK) or 1/2000 anti-CD68 (Abcam). Subsequently, slides were incubated with a one-step polymer-horseradish peroxidase (EasyPath-Erviegas, São Paulo, Brazil) for 20 min, developed with 3, 3′-diaminobenzidine chromogen (Sigma-Aldrich), and counterstained with Harris hematoxylin. Semi-quantitative analysis was performed using Image-Pro Plus 4.5 software (Media Cybernetics) in 10 randomly selected fields (20× objective for α-SMA and 40× objective for CD68) per section from the left lobe. Data analysis was based on the following calculations [α-SMA^+^ cells area (%) = α-SMA^+^ area/(total field area − vascular luminal area)] and [CD68^+^ density = CD68^+^ cells/total field area (mm^2^)].

### 4.8. Analysis of Serum Biochemical Parameters

Serum levels of ALT (IU/L), AST (IU/L), conjugated and total bilirubin (mg/dL) and albumin (g/dL) were measured with an automated spectrophotometric Labmax 240 analyzer (Labtest Diagnostica, Vista Alegre, Brazil).

### 4.9. Analysis of Hepatic Anti-Oxidant Enzymes

The activity of SOD, GPx, GR, and catalase was assayed in mouse liver tissue, as previously described [43]. SOD activity was calculated by interpolation of the percentage of inhibition of formazan generation using a linear regression curve prepared with SOD from bovine erythrocytes (Sigma-Aldrich). GPx activity was continuously monitored at 340 nm absorbance over 4 min at 37 °C by the detection of nicotinamide adenine dinucleotide phosphate. GPx activity was calculated by linear regression using the percentage of inhibition promoted by GPx (Sigma-Aldrich). GR activity was determined in the liver homogenate at an absorbance of 340 nm over 26 min at 37 °C by the measurement of nicotinamide adenine dinucleotide phosphate oxidation. GR activity was calculated based on linear regression using the percentage of inhibition triggered by GR (Sigma-Aldrich). Catalase activity was continuously monitored in liver homogenate at 240 nm absorbance over 8 min at 30 °C by the measurement of hydrogen peroxide. A standard curve was prepared using catalase enzyme (Sigma-Aldrich). Enzymatic activity was expressed in U/mg protein.

### 4.10. Analysis of Liver Inflammatory Markers

Inflammatory markers were evaluated in hepatic homogenates from mouse using a mouse inflammation antibody membrane array for 40 targets (ab133999, Abcam), following the manufacturer’s instructions. Briefly, antibody spotted membranes were blocked during 30 min at room temperature. Thereafter, blocked membranes were exposed to 250 μg protein/ml blocking buffer overnight at 4 °C while shaking. Membranes were washed and incubated with biotin-conjugated anti-cytokines for two hours at room temperature. Then, membranes were washed and exposed to horseradish peroxidase-conjugated streptavidin for two hours at room temperature. Following washing, proteins were detected by means of enhanced chemiluminescence and visualized by Chemidoc^TM^ MP system (Bio-Rad, Hercules, CA, USA). Semi-quantitative results were obtained after densitometric analysis. Thereafter, background was subtracted from each data point and results were normalized against the positive controls provided by biotin-conjugated IgG and the results were presented as relative signal intensity against saline.

### 4.11. Statistical Analysis

The number of biological (*n*) and technical (*N*) repeats for each type of analysis varied and is specified in the discussion of the results. All data were expressed as mean ± standard error of the mean (SEM). Data distribution was determined by D’Agostino-Pearson normality test for large n or Shapiro-Wilk normality test for low *n*. Thereafter, results were statistically processed by 1-way analysis of variance (ANOVA) followed by *post hoc* Bonferroni correction using GraphPad Prism7 software (version 7, GraphPad Software, La Jolla, CA, USA) with probability (*p*) values of less than or equal to 0.05 being considered as significant.

## Abbreviations

ALT	Alanine aminotransferase
ANOVA	Analysis of variance
α-SMA	Alpha smooth muscle actin
AST	Aspartate aminotransferase
ATP	Adenosine-5’-triphosphate
CBX	Carbenoxolone
Cx	Connexin
DF	Divalent free
FRAP	Fluorescence recovery after photobleaching
GJ(s)	Gap junction(s)
GM-CSF	Granulocyte-macrophage colony stimulating factor
GPx	Glutathione peroxidase
GR	Glutathione reductase
HBSS	Hank’s balanced salt solution
HSC(s)	Hepatic stellate cell(s)
IL	Interleukin
KC	Growth-regulated alpha protein
LIX	Lipopolysaccharide-induced CXC chemokine
*n*	Number of biological repeats
*N*	Number of technical repeats
*p*	Probability
SEM	Standard error of the mean
SOD	Superoxide dismutase
TAA	Thioacetamide
TAT	Transactivator of transcription
TECK	Thymus-expressed chemokine
